# Tracing sources of inorganic suspended particulate matter in the Great Barrier Reef lagoon, Australia

**DOI:** 10.1038/s41598-024-66561-5

**Published:** 2024-07-08

**Authors:** Zoe T. Bainbridge, Jon M. Olley, Stephen E. Lewis, Thomas Stevens, Scott G. Smithers

**Affiliations:** 1 Catchment to Reef Research Group, TropWATER, James Cook University, Townsville, 4811 Australia; 2 Australian Rivers Institute, Griffith University, Nathan, Brisbane, 4222 Australia; 3 Earth and Environmental Sciences, College of Science and Engineering, James Cook University, Townsville, 4811 Australia

**Keywords:** Sediment tracing, Fine sediment, Rare earth elements, Catchment to marine continuum, Terrigenous sediment, Subsoils, Suspended particulate matter (SPM), Environmental chemistry, Environmental monitoring, Geochemistry

## Abstract

Water clarity on the inshore Great Barrier Reef (GBR) is greatly influenced by terrestrial runoff of suspended particulate matter (SPM). Catchment sediment tracing studies often do not extend into the marine environment, preventing the analysis of preferential marine transport. This study employs novel collection and sediment tracing techniques to examine the transport of the terrigenous ‘mineral’ component of plume SPM within the GBR lagoon for two flood events. Utilising geochemical, radionuclide and clay mineral analysis, we trace terrigenous mineral sediments > 100 km from the river mouth. We show that the SPM geochemistry is highly influenced by particle-size fractionation, desorption, and dilution within the plume, rendering traditional tracing methods unviable. However, the ratios of rare earth elements (REE) to thorium (Th) provide stable tracers of mineral SPM transported across the catchment to marine continuum and allow the identification of discrete catchment sources for each flood event. Plume sediment radionuclides are also stable and consistent with sub-surface erosion sources.

## Introduction

Excess fine suspended particulate matter (SPM) is a major contributor to the declining health of the world’s largest coral reef ecosystem, the Great Barrier Reef (GBR)^1–4^. The most damaging SPM that causes prolonged reductions in water clarity is delivered during river floods when flood plumes disperse into the marine environment^5–7^. These plumes can extend hundreds of kilometres offshore and may reduce water quality over large areas of the GBR lagoon^3,8,9^. The excess SPM comprises terrigenous mineral particles and organic materials originating from either the river or produced within the plume^3,7,10^. While the organic material is rapidly processed and recycled in the marine environment^10–12^, the terrigenous mineral particles often form the nucleus of sediment flocs^7,13^ and hence ‘control’ water clarity in the GBR. Identifying the dominant sources of this material is critical to prioritise remediation programs, worth $100 M’s, aimed at protecting the reef ecosystems^1,14^. A major knowledge gap hindering this endeavour is understanding which components of the terrigenous mineral particles are preferentially transported furtherest within the GBR lagoon, and identifying both the dominant erosion processes and the specific spatial source areas within the catchments. While two previous studies presented some evidence for the preferential transport of SPM in GBR flood plumes^15,16^, the SPM which likely has the greatest influence on water clarity in the offshore plume waters has not been characterised as sufficient sample mass could not be collected. Here we use novel collection and tracing methods to examine the origins of the terrigenous mineral component of plume SPM within the offshore waters and provide new insights into sediment transport processes within the GBR lagoon.

**Figure 1:**
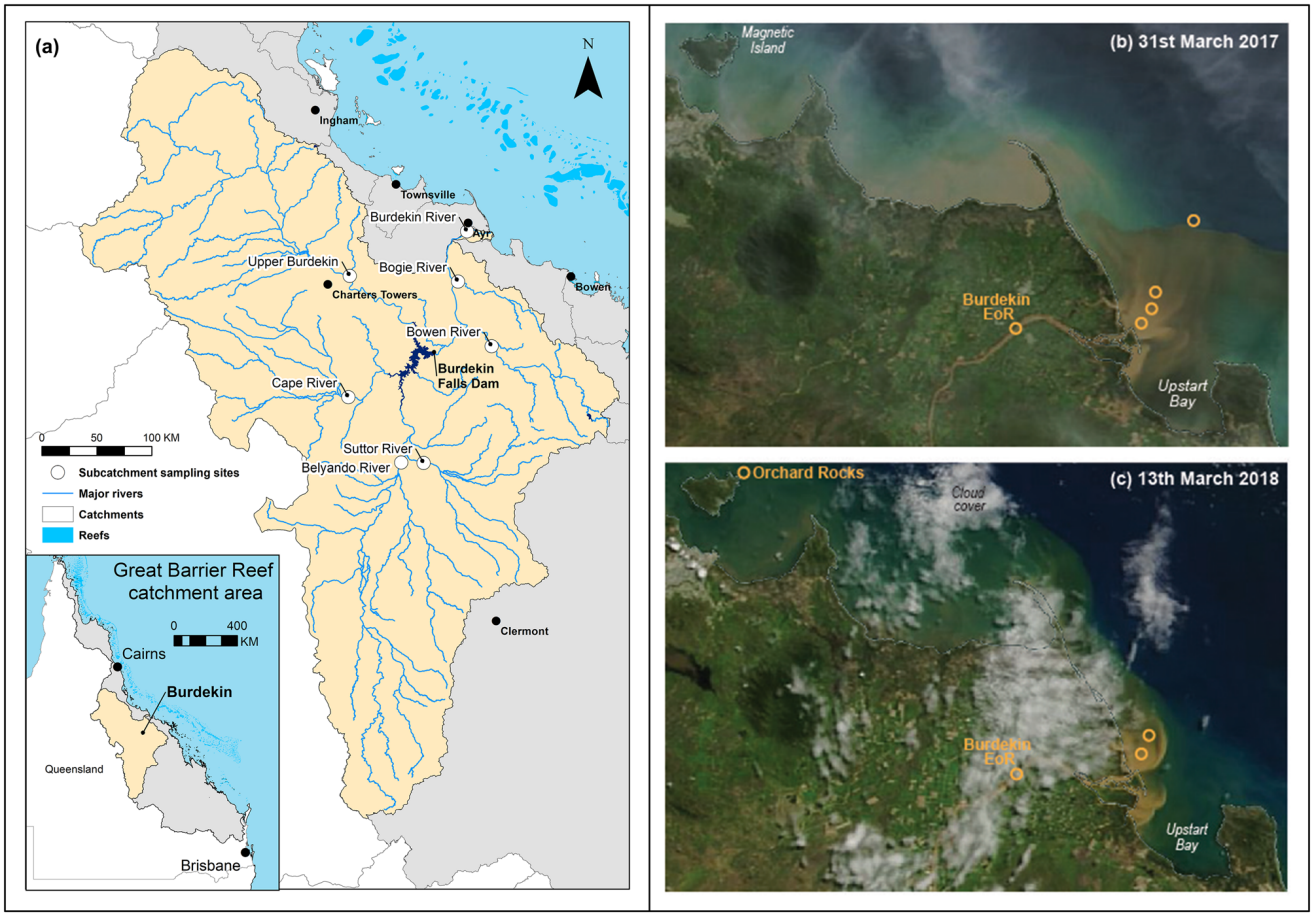
Map of the Burdekin river catchment (**a**) and six sub-catchment sampling sites, Burdekin Falls Dam and end of-river sample site locations (white circles). MODIS satellite imagery of the 2017 (**b**) and 2018 (**c**) Burdekin river flood plumes captured following peak river discharge, including sampling locations on the 31st March 2017 (**b**), 6th March 2018 in Upstart Bay and 13th March at the northern Orchard Rocks site (**c**) (Imagery source: NASA MODIS (Terra)).

The GBR extends offshore from the northeastern Australian coastline over a stretch of ~ 2000 km (Fig. 1). The adjoining coastal catchments cover an area of 423,000 km^2^, all of which drains into the lagoon located between the coast and the outer reefs. The Burdekin River catchment (130,000 km^2^) which drains into the central region of the GBR is the largest single contributor of terrestrial SPM to the GBR lagoon accounting for, on average, ~ 40% of the total annual load^17^. Sediment budgets show two major sediment source areas in the Burdekin catchment: the area below the Burdekin Falls Dam (BFD) and the Upper Burdekin sub-catchment^18^. During large Burdekin River flood events, the riverine flood plumes can cover large areas of the inshore and mid shelf sections of the GBR lagoon and impinge on high value seagrass meadow and coral reef habitats^4,19,20^. This study focuses on two Burdekin River flood plumes that occurred in March 2017 and March 2018 (Fig. 1b,c).

SPM in the river plumes originates from the erosion of catchment surface soils and from gully and stream bank erosion (channel erosion). At the local level, it is common for either surface soil or channel erosion to be the dominant sources. The management of these two erosion types differs. Channel erosion is best managed by preventing stock access to streams, protecting riparian vegetation cover in areas prone to channel erosion, revegetating bare banks, and reducing sub-surface seepage in areas with erodible subsoils. Surface soil erosion is best managed by promoting groundcover, maintaining soil structure, and promoting deposition of eroded sediment before it reaches the stream. It is therefore important to be aware of the dominant type of erosion before designing and implementing catchment intervention measures. Studies using fallout radionuclide concentrations (^137^Cs, ^210^Pb_ex_ and ^7^Be) have shown sub-surface erosion dominates (> 80%) the supply of sediment to rivers draining into the GBR lagoon^21–26^. As a result, management efforts are currently focussed on controlling sub-surface erosion. However, fallout radionuclide analysis has been limited in flood plumes and consequently the dominant erosion processes generating the SPM preferentially carried within the offshore plume waters has yet to be determined.

As SPM moves offshore within riverine flood plumes it is subjected to major transformations including flocculation, preferential deposition (i.e. particle size/mineralogy related), desorption/adsorption of elements and the addition of new components such as microbes (bacteria/fungi) and algae (phytoplankton/zooplankton)^7,10,13,15,27–31^. For the larger rivers that deliver the highest SPM loads to the GBR (e.g. Burdekin and Fitzroy) more than 80% of the terrestrially derived material is deposited near the river mouth and retained^3,28,32,33^. Only the fine and less dense components (clay, fine silt fractions and organic flocs) travel further in the flood plumes^7,13^. To improve GBR water quality this is the material that must be characterised and for which source areas must be identified and managed^3^. However, tracing specific spatial source areas in catchments, and identifying the key erosion processes generating this fine and less dense material, is a major challenge. In this study we use a novel high-volume filtration system^34^ to capture sufficient SPM mass from low concentration flood plume waters (i.e. < 10 mg/L) to enable catchment source tracing for the first time.

To be useful in tracing the source of sediments^35,36^, a parameter needs to be conservative and:

- For a given source of sediment, which does not change with respect to time, a sediment tracer signal must also be constant in time or vary in a predictable way.
- For a given source of sediment, which does not change with respect to distance along a transport path, a sediment tracer signal must also be constant along this path or vary in a predictable way.
- The tracer signal should allow sediments derived from different source areas to be distinguished.

The properties of sediments (e.g. mineralogy, major and trace element geochemistry, radionuclides, isotopes, particle size) can be used to determine its source provided it fulfills the requirements outlined above^15,16,24,35^. Here we use sediment geochemistry and clay mineralogy to determine the provenance of the mineral component of the plume SPM and fallout radionuclide activity concentrations to identify the dominant erosion process.

## Results and discussion

### The flood events

The March 2017 flood was associated with heavy rainfall and rapid river rises from Tropical Cyclone Debbie’s crossing of the north Queensland coastline. The Burdekin River discharged 2.49 million ML during this event with 65% of this flow measured during a peak six-day period between the 29^th^ of March and the 3^rd^ of April. An estimated 1.5 million tonnes of suspended sediment were exported during this six-day event^37^. This discharge was almost exclusively sourced from the catchment area below the BFD, including the Bowen and Bogie Rivers. The resultant flood plume moved offshore in an easterly/south-easterly direction in response to the northerly winds (Fig. 1b). A SPM sample was collected from the Burdekin River flood peak at the End of River (EoR) Inkerman Bridge site on the 31st of March, together with four samples collected along a salinity gradient of < 1, 6, 12, and 26 practical salinity units (PSU) across the flood plume (hereafter referred to as plume samples) (Fig. 1b).

In late February/early March 2018, heavy rainfall in the Upper Burdekin River sub-catchment, triggered minor flood levels in the downstream river reaches. A total of 4.87 million ML was discharged during this event, which was dominated by run-off from the Upper Burdekin River sub-catchment. Satellite imagery showed the early plume extent was largely confined to Upstart Bay, and began to extend northwards into Bowling Green Bay on the 6th of March. By the 10th of March the plume had extended northwards along the coastline past Magnetic Island and into the Palm Island Group and the plume continued to influence this northern region until at least the 29th of March (Fig. 1c). Unfortunately, poor weather restricted SPM sampling from this plume. However, two SPM samples were collected near the river mouth in Upstart Bay on the 6th of March (at 4 and 23 PSU) with an additional surface water sample collected at Orchard Rocks off Magnetic Island, 115 km from the river mouth on the 13th of March (Fig. 1c). Four samples were also collected through the flood-peak capturing SPM on the rising, peak and falling stages at the EoR Inkerman Bridge site. For comparison a sample was also collected during the 2019 flood plume from the Orchard Rocks sampling site.

### Flood event catchment sediment sources

To identify the sediment source of the Burdekin EoR samples we compared their geochemistry to a suite of samples collected from the six major Burdekin sub-catchments (Upper Burdekin n = 22, Belyando n = 10, Cape n = 8, Suttor n = 13, Bowen n = 11, Bogie n = 7). The nonparametric Kruskal–Wallis H-test was first used to test the ability of each element to distinguish between each of the sediment source groups. Elements which passed this test were then used in linear discriminant analysis to identify the best combination of elements which could classify each sample back to its original source. The combination of K_2_O, TiO_2_, Ce, Co, Cr, La, Th, Y, Ni, Rb, Ba, Dy, Pr, and Er were able to classify 100% of the source samples back to the six source sub-catchments. These elements were then used (after normalisation of the concentrations) in agglomerative-hierarchical-clustering to identify the closest geochemical associations of the EoR samples. The 2017 EoR sample closely matched samples from the Bowen river (see Supplementary Material) and all the 2018 EoR samples closely matched samples collected from the Upper Burdekin sub-catchment. Hence traditional tracing methods were able to confidently attribute fine sediment sources within the Burdekin River catchment and the sources were in close agreement with the areas where flooding occurred.

### Proportion of organic matter, SPM concentrations and evidence of fractionation

**Figure 2:**
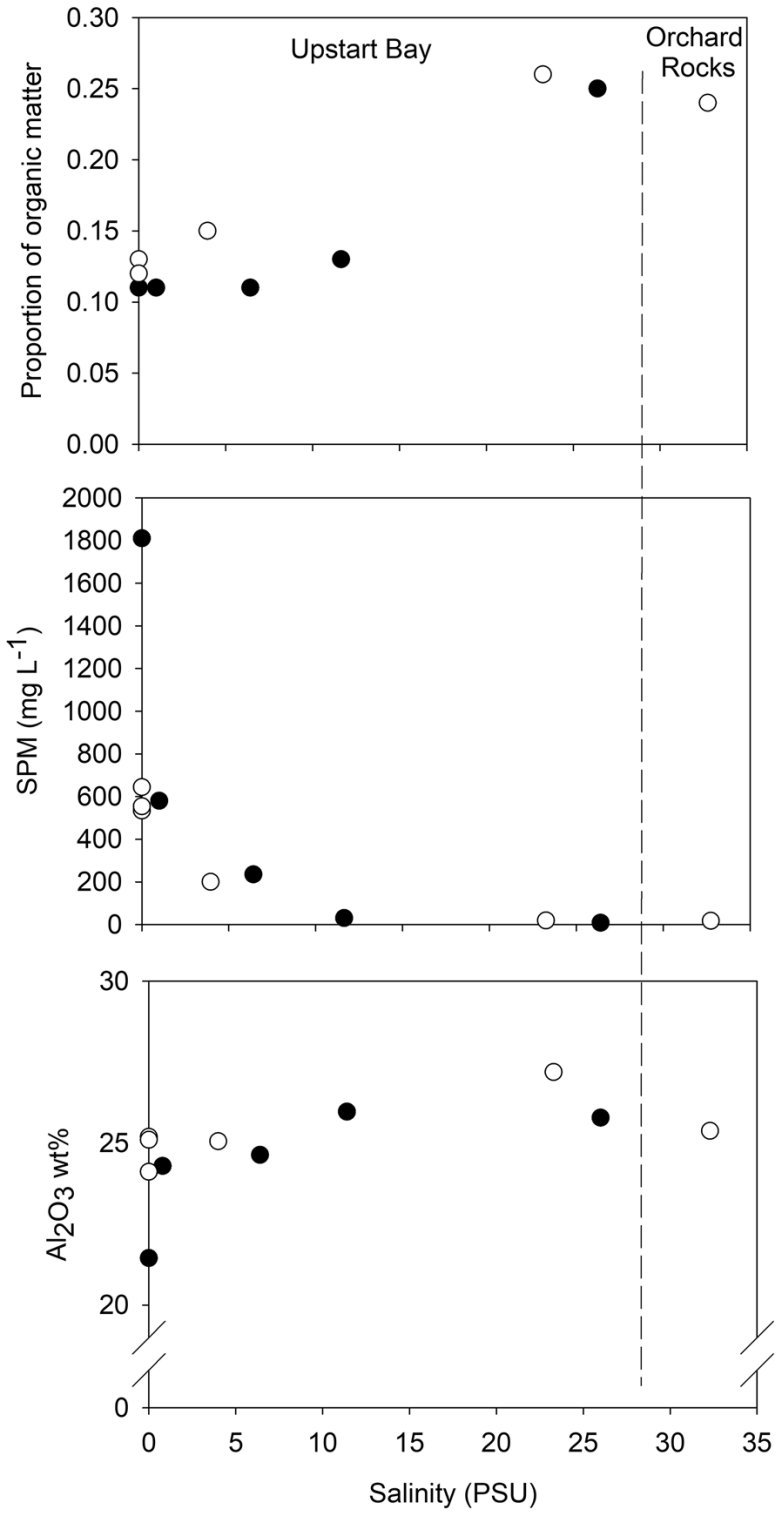
Proportion of SPM organic matter (%), SPM concentration (mg/L) and Al_2_O_3_ concentrations (wt%) versus salinity in samples collected from EoR, Upstart Bay and at Orchard Rocks during the 2017 (closed circles) and 2018 (open circles) flood plume events.

In both the 2017 and 2018 events, the particulate organic components of the SPM at the EoR freshwater site (0 PSU) ranged between 11 and 13% (Fig. 2). Hence, the terrigenous, mineral component dominated SPM EoR export (535–1800 mg/L), with 82–93% of mineral sediment grains < 20 µm^7^.

SPM concentrations in the plume samples declined from ~ 200 mg/L at ~ 5 PSU to < 20 mg/L by the mid-salinity range (i.e. 14 to 25 PSU), consistent with previous observations^3,5,7^. The 0 to 15 PSU ‘primary estuarine’ zone coincides where the bulk SPM delivered by the Burdekin River is deposited within Upstart Bay. The proportion of SPM organic matter ranged from 11–21% across these samples and increased with salinity. Concentrations of Al_2_O_3_ (wt% oxide) in the inorganic fraction of the SPM increased along this salinity gradient (Fig. 2). This increase indicates an enrichment in the clay component of the terrigenous SPM that remains in the water column.

**Figure 3:**
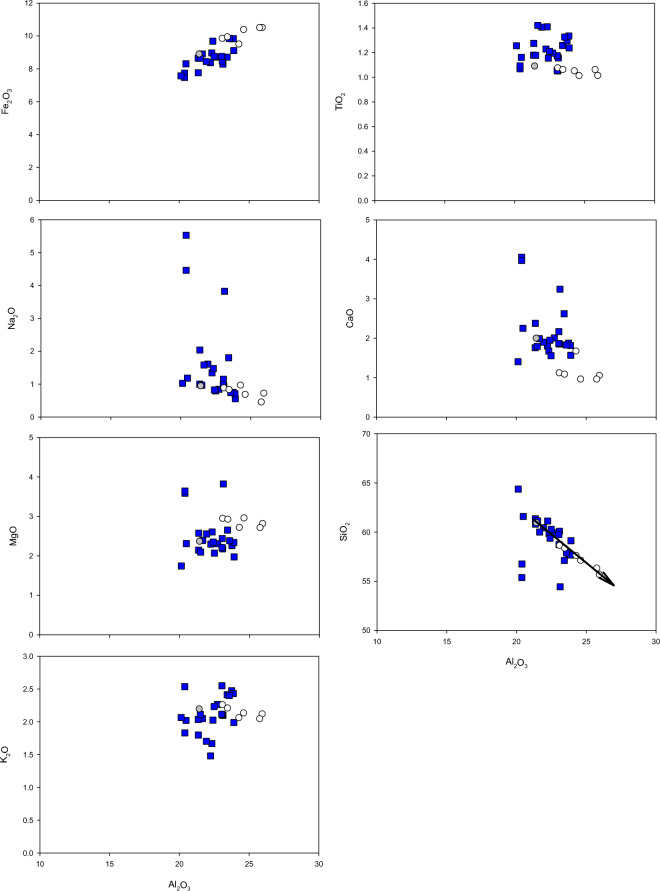
Major element concentrations in SPM samples collected from Bowen-Bogie river (blue) sub-catchments (Fig. 1) shown together with the EoR (grey) and flood plume (white) samples for the 2017 event. Element concentrations (weight percent oxides) are plotted against Al_2_O_3_ (wt%). The SiO_2_ to Al_2_O_3_ plot arrow indicates expected changes in concentrations due to mineral clay enrichment.

This enrichment of the clay component also coincides with a marked variation of the SPM geochemistry (Fig. 3). The March 2017 floodwaters were almost exclusively sourced from the Bowen-Bogie river sub-catchments. It would therefore be expected that the EoR and plume SPM samples would similarly be derived from these sub-catchments, and that the major element concentrations of these samples would fall within the range of samples collected from these sources. While the EoR sample closely matched the Bowen sub-catchment source, the adjacent plume samples collected from Upstart Bay fell well outside the source range for this catchment area. Hence the traditional tracing approaches would suggest that the plume SPM would have a different source to that measured at the EoR (Supp. Material; see also^38^).

**Figure 4:**
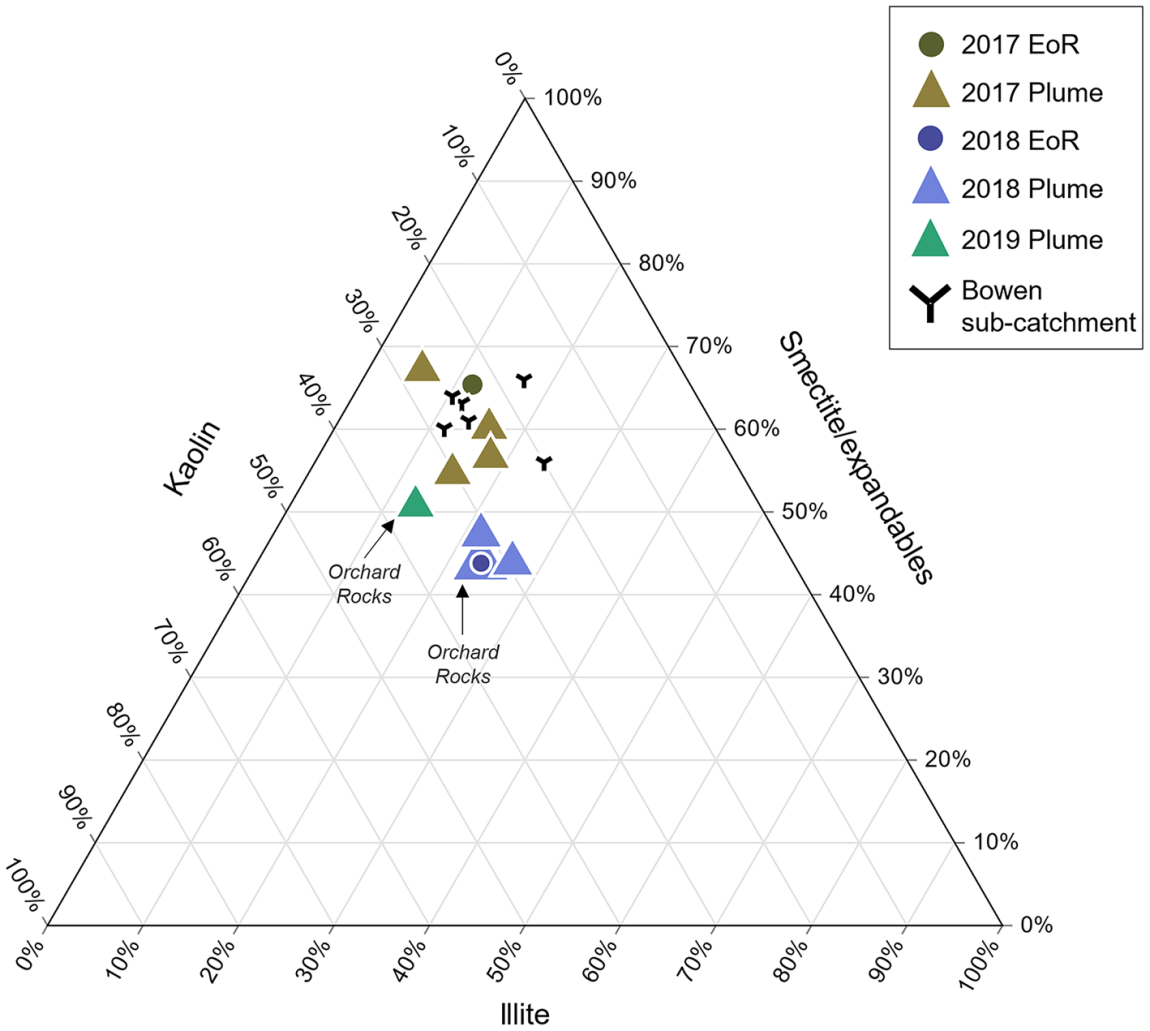
Ternary diagram showing the proportion of kaolin, illite and smectite/expandable clays for 2017 and 2018 events in the Burdekin EoR and flood plume sediment samples. The Bowen sub-catchment source samples are also shown for comparison to the 2017 EoR and plume samples, as well as a 2019 plume sample collected at Orchard Rocks.

Further inspection of the plume SPM geochemistry data show that changes in SiO_2_ and Al_2_O_3_ are tightly correlated (see arrow on the plot in Fig. 3) and follow the expected concentration changes that result from an enrichment in mineral clays with the corresponding loss of quartz and feldspar minerals. This result suggests that changes in the major element concentrations between the Bowen source samples, the EoR samples and the plume samples are the result of particle-size fractionation during transport. Quantitative clay mineralogy analysis for the 2017 and 2018 event SPM samples also shows an increase in the proportion of finer smectite/expandable clays from the EoR to plume sediment samples at the expense of larger illite particles (Fig. 4). Hence both the major element geochemistry and clay mineralogy data clearly indicate that the plume sediment samples have been affected to varying degrees by particle-size fractionation. In that regard, the traditional geochemical sediment tracing approaches, using either major or trace element concentrations in a mixing model (e.g.^35,39–41^) cannot be used to determine sediment sources and a new approach is warranted.

### Determining the provenance of the terrestrial component of SPM

The concentrations of rare earth elements (REE) plus yttrium (Y), scandium (Sc) and thorium (Th) have been widely used in tracing studies^16,42–45^ because they strongly partition into the mineral particulate phase, they behave coherently during weathering, erosion, and fluvial transportation, and they are highly resistant to chemical mobilisation. In the GBR lagoon they will also be primarily derived from the terrestrial environment^46^ and therefore provide a means of tracing the terrestrial component of the flood plume SPM. While concentrations of these elements will also be affected by particle-size fractionation, it is likely, because of their chemical stability and affinity for clay minerals, that element ratios will remain stable throughout transport across the catchment and marine environments. The element ratios should also be unaffected by the addition of marine carbonate or biogenic silica because these marine derived components have negligible concentrations of these elements.

**Figure 5:**
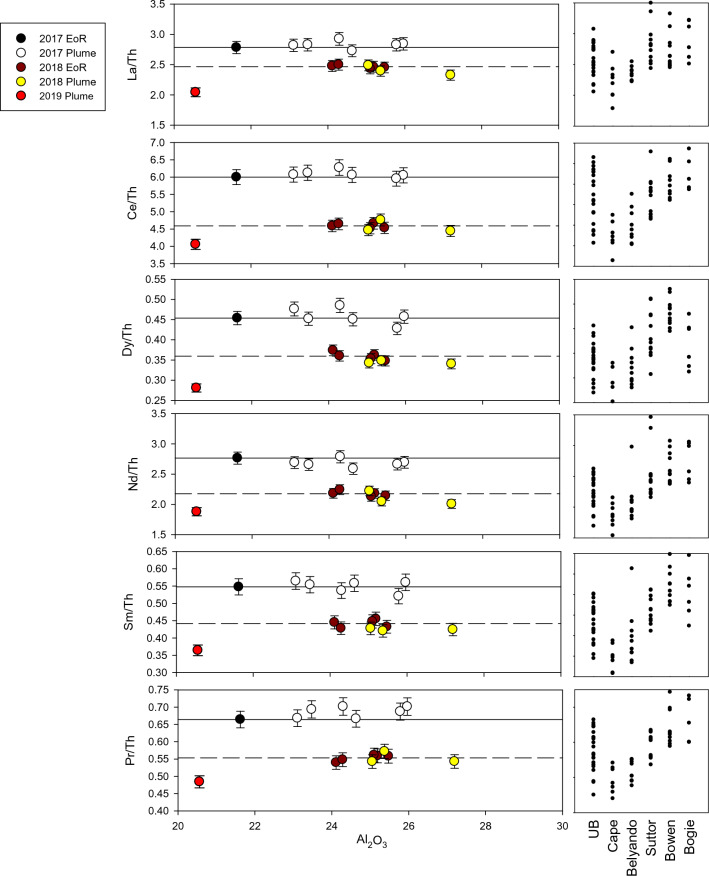
Selected rare earth element to Thorium ratio vs Al_2_O_3_ (wt%) plots for the EoR and plume sediment samples during the 2017 and 2018 events (left panel). The mean ratios of EoR samples are represented by solid (2017) and dash (2018) lines, respectively. Sub-catchment sediment sample ratios are shown for comparison on the right panel. Error bars (estimated from repeat sample analyses) are equivalent to one standard error. For comparison a sample collected during the 2019 flood plume from the Orchard Rocks sampling site is also shown (red dot).

In both the EoR and plume SPM, Al_2_O_3_ wt% concentrations respond to differences in clay mineral composition associated with changing geological source areas^15^ (Fig. 4), the preferential loss of other components, such as quartz and feldspar (i.e. increase in Al_2_O_3_), or the gains of components such as marine carbonate and biogenic silica (i.e. decrease in Al_2_O_3_). In Fig. 5 selected REE/Th ratios for EoR and plume sediment samples collected during the 2017 and 2018 flood events are plotted against Al_2_O_3_ (wt%). The solid lines and dashed lines represent the mean ratios for the 2017 and 2018 EoR samples, respectively. The corresponding sediment sample ratios from the Burdekin sub-catchments are shown on the right-hand side of the plot for comparison. All of the REE/Th ratios plus Sc and Y from each of the 2017 and 2018 events remain consistent against changing Al_2_O_3_ concentrations with the individual ratios being different between the 2017 and 2018 events. Importantly, the REE/Th, Sc/Th and Y/Th ratios from the 2017 and 2018 plume sediments were consistent with those from their respective EoR samples despite the significant changes in major element concentrations and mineralogy measured across the salinity gradient. This result indicates that these ratios are unaffected by particle-size fractionation during sediment transport and thus faithfully record the original source areas of the SPM as it is transported across the catchment and marine environments. Further, the consistency of the ratios between the EoR and plume SPM in each year indicates a common source, and the difference in ratios between years indicates that sediments from the 2017 and 2018 events were derived from different source areas within the catchment.

The remarkable stability of the REE/Th ratio and the clay mineralogy signature of the SPM samples across the plume salinity gradient is shown by the sample collected from Orchard Rocks, 115 km from the river mouth. This sample, taken from 32 PSU (i.e. ~ 10% freshwater) during the 2018 event, exhibits highly consistent ratios matching the EoR sample and hence the Upper Burdekin sub-catchment source. Interestingly, a surface SPM sample collected from this same location during the 2019 flood plume displayed considerably different REE/Th ratios than the SPM measured in the 2018 flood plume (Fig. 5). This result not only highlights the transient sources of SPM in river plumes but also demonstrates that our method can reliably identify different catchment sources of SPM that have travelled considerable distances in the marine environment. The SPM in the 2019 flood plume may have been derived from a complex array of potential catchment sources including within the Burdekin (Upper Burdekin), Haughton and Ross basins, all of which experienced major flooding coinciding with the sample collection. Additional tracing data from these catchments is required to determine the source of the SPM from the 2019 plume.

### Erosion processes

**Figure 6:**
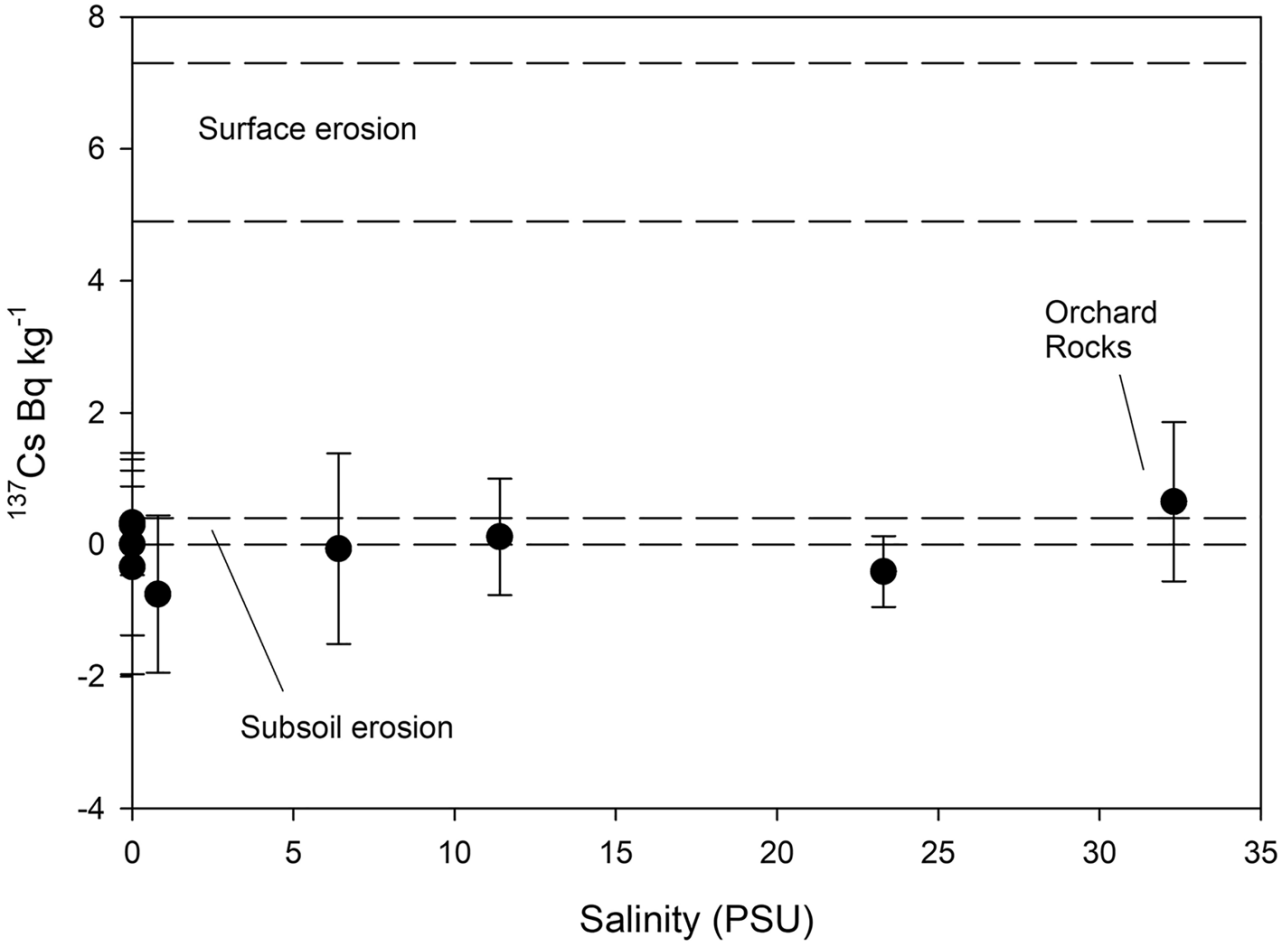
Fallout radionuclide ^137^Cs activity concentrations measured in EoR and in plume samples from the 2017 and 2018 events against salinity (PSU). Ranges, at two standard errors of the mean, for surface and sub-surface erosion are also shown. Error bars represent measurement uncertainties at one standard error of the mean.

The fallout radionuclide ^137^Cs is concentrated in the surface soil^25,26^. Consequently, sediments derived from surface soil erosion will have high ^137^Cs concentrations, while sediment eroded from subsoils, by gully or channel erosion will have little or no fallout radionuclide present. The values of 6.1 ± 1.2 and 0.2 ± 0.2 Bq Kg^−1^ (error at 2σ) for surface and sub-surface erosion, respectively (after Wilkinson et al.^26^) were used to compare our results (Fig. 6). Activity concentrations measured in EoR and plume samples from the 2017 and 2018 events are all consistent with sources almost exclusively from sub-surface erosion (Fig. 6). It is noteworthy that fractionation by particle size, which preferentially transports the smaller particles, would be expected to increase ^137^Cs concentrations in the plume samples as it is more strongly bound to finer particle surfaces as well as the greater surface area per unit weight of the smaller particles. Despite this expectation, our data show that the activity of the ^137^Cs radionuclide does not significantly change over the salinity gradient and strongly signifies the dominance of sub-surface erosion processes.

### Summary and implications

In both the 2017 and 2018 flood events the terrigenous-mineral component dominates the SPM export from the Burdekin catchment (> 85%) and it remains the dominant component (> 70%) in flood plume waters 115 km from the Burdekin River mouth, despite the addition of marine organic matter. While the organic material in the offshore plume waters is rapidly processed and recycled (and more closely resembles a marine composition)^10–12^, our findings unequivocally show that the terrigenous mineral particles retain their key characteristics and can be traced back to the sub-catchments from which they were derived. Changes in these SPM characteristics between events indicates that, in each case, the sediments were derived from a different sub-catchment source. In both the 2017 and 2018 events the characteristics of plume SPM were consistent with the sub-catchments from which the flood waters originated. In both events, however, sub-surface erosion dominates (> 90%) the supply of material to the EoR and into the plume. This study clearly demonstrates that terrigenous sediments eroded from subsoil sources in the Burdekin catchment are being transported in offshore plume waters > 100 km from the river mouth. Previous studies have documented the extensive (> 100 km) reach of the Burdekin River plume in the GBR lagoon through salinity readings, water quality measurements, microscopic observations and luminescent line markers in coral cores^5,7,13,47,48^. However, this study has been the first to trace the mineralogical and geochemical composition of the SPM in these far reaches of the plume back to a source within the Burdekin catchment. Whilst particle-size fractionation occurs during sediment transport, the relationship between the rare earth elements and thorium (REE/Th) ratios and clay mineralogy signatures are robust and clearly enable terrigenous sediment to be traced across the catchment to reef continuum. This study provides the first characterisation of the SPM likely to most reduce water clarity in offshore plume waters in the GBR, and provides a new approach to trace SPM erosion processes and catchment sources. The new method eliminates the earlier barriers associated with particle-size fractionation, desorption, addition of organics and biogenic materials^13,15,29,38,49^. Our findings support current management efforts which are focused on controlling sub-surface erosion in the Bowen sub-catchment but also points to the importance of the other major source area to manage, the Upper Burdekin sub-catchment. Historically, some of the largest Burdekin flood events (both in volume and plume spatial extent) have been driven by this Upper Burdekin sub-catchment which contributes the greatest volume of water and sediment load during these very large events^13,18^. Future work could extend this research to other river flood plumes as well as to trace the terrigenous origins of sediments deposited and resuspended within marine environments.

## Methods

### Sample collection

#### Flood plume samples

The SediPump® high-volume filtration system^34^ was used to capture representative and sufficient SPM for sediment tracing analysis from low concentration (i.e. < 10 mg L^−1^) plume waters^7^. The SediPump® allows large volumes of water (> 6 KL in ~ 2 h) to be filtered through a nominal 1 µm string filter to recover 2–5 g of material. The SediPump® was run between 30 min and 3 h (depending on the SPM concentration of the water and field time constraints). Following collection, the string filters were placed into a 10 L container with water from the collection site and, on return to the laboratory, cut to release the SPM. The samples were transferred into dialysis tubing and salts were removed through continuous rinsing in RO water^7^. The < 10 µm fraction was then recovered via the settling method (described in Bainbridge et al.^15^) for geochemistry, clay mineralogy and radionuclide analyses. In the case of two plume samples from the 2018 event, the samples were sieved through a 38 µm mesh and this fraction was recovered due to the smaller mass of material. The recovered samples were oven-dried at 60 °C and homogenous sub-samples prepared for the various tracing analyses.

#### End-of-river samples

During each river flow event (March 2017 and March 2018) EoR suspended sediment samples (freshwater) were collected from the Burdekin River at the Inkerman Bridge. Surface water ‘grab’ samples (top 0.5 m of water column) were collected from the well-mixed centre channel for each year’s event and represent the material being delivered to the river mouth. The < 10 µm sediment fraction of each discrete sample was recovered, including one sample from the March 2017 flood peak, and four samples from the rising (replicate set), peak and falling hydrograph of the March 2018 event.

#### Major tributary sub-catchment samples

River water samples were also collected to characterise each of the major contributing sub-catchments of the Burdekin basin (Fig. 1). Event samples were collected at streamflow gauge locations draining five of the six main sub-catchments (Upper Burdekin (n = 22); Cape (n = 8); Belyando (n = 10); Suttor (n = 13); and Bowen (n = 11)) and also from the ungauged Bogie River (7 discrete and composite samples) collected over five consecutive wet seasons (2005/06–2009/10). Further sampling and site location details are provided in Bainbridge et al.^18^ and a subset of Bowen samples in Wilkinson et al.^50^. The < 10 µm sediment fraction was recovered for each of these samples for geochemical and clay mineralogy analysis.

#### Suspended particulate matter and organic matter contents

Suspended particulate matter and organic matter concentrations of the samples were collected in a 10 L container at the surface of the water column (upper 50 cm) and a well-mixed aliquot was then transferred into a 1 L bottle. The water samples were kept refrigerated (4 °C) prior to analysis.

### Sample analysis

#### Geochemistry

The five EoR and ten plume samples were analysed at the Queensland Government Department of Environment, Science and Innovation (DESI) Chemistry Centre (Brisbane, Australia). As described in Bainbridge et al.^38^, the dried < 10 µm samples were fused with lithium metaborate flux at 975 °C and analysed by Inductively Coupled Plasma-Optical Emission Spectrometry (ICP-OES) for the major element concentrations and Inductively Coupled Plasma-Mass Spectrometry (ICP-MS) for the trace and rare earth element (REE) concentrations. These instruments were calibrated with certified commercial single- and multi-element standard solutions (9 for ICP-OES and 10 for ICP-MS).

The 71 sub-catchment samples were analysed at the Advanced Analytical Centre (AAC), James Cook University (Townsville, Australia). The dried < 10 µm samples were fused with lithium metaborate flux at 1050 °C to produce a glass disc and analysed by laser ablation ICP-MS for both major and trace element concentrations following Eggins^51^. Two certified standard reference materials (NIST610/612) were used for spectrometer calibration.

Interlaboratory comparisons were conducted using reference standards and showed good agreement for both major and trace element data. The measured major element concentrations were converted to weight percent oxides and summed. The summed weights were then normalised to 100% (i.e. exclude the loss on ignition). The same correction factors were applied to the trace element data.

#### Fallout radionuclides

The < 10 µm fraction of five EoR and five flood plume samples were analysed for ^137^Cs by high-resolution gamma-ray spectrometry following the procedures of Leslie^52^. Samples ranged in weight from 1.2 to 13 g and were counted for a minimum of three days on a high-resolution germanium gamma detector at Griffith University. Measurement uncertainty is represented as one standard error of the mean, incorporating analytical detection limits for each radionuclide.

#### Clay mineralogy

Clay mineral analysis was conducted on the < 10 µm fraction of two EoR, nine plume samples and five samples from the Bowen sub-catchment. Oriented clay smears were analysed by x-ray powder diffraction (XRPD) at the James Hutton Institute, Scotland, with each sample repeatedly run on the diffractometer following various treatments (i.e. air-dried, ethylene glycol, multiple heating) to identify unique clay mineral diffraction patterns^53^. The reference intensity ratio (RIR) was used to determine the relative weight percentages of the clay mineral groups kaolin, illite and ‘expandables’. The expandable clay group includes smectites (e.g. montmorillonite) as well as other mixed-layer clay minerals with a smectitic (expandable) component, all of which have a high capacity to ‘shrink-swell’ through water and cation exchange^15^. As described in Bainbridge et al.^15^, this classification emphasises that the expandable clays are not necessarily pure smectites and may vary in composition from place to place. Kaolin is used in preference to kaolinite since the kaolin group mineral halloysite is likely present in some sources and we have not attempted to distinguish between kaolinite and halloysite in this investigation.

#### Suspended particulate matter (SPM) and organic matter

SPM, measured as total suspended solids concentrations, were determined using standard gravimetric filtration (Method 2540D^54^). Known volumes of well-mixed samples were vacuum filtered onto pre-weighed 0.4 µm polycarbonate filter papers which were well-flushed of salts using RO water and dried at 103–105 °C for 24 h and reweighed. The organic matter contents were determined using APHA Method 2540E^54^ for volatile solids, where ~ 0.1 g of pre-weighed sample was ignited to 550 °C for 4 to 5 hours^7^. Sample weight lost during ignition represents the volatile solids component, an approximation of the organic matter content.

### Determining the provenance of sediments

#### Geochemistry

To determine sediment provenance, we compared the geochemical characteristics of SPM samples collected from Burdekin EoR and plumes in 2017 and 2018, with samples collected from the major Burdekin sub-catchments. The geochemical characteristics of sediments are strongly influenced by those of the soils from which they are derived^36,55–57^. Sediments initially maintain these distinctive geochemical properties relating to their genesis during sediment generation and transport processes^22,55^, although physical (e.g. particle size) and biochemical (adsorption/desorption, dilution) processes may need to be accounted for during transport^49^. The effects of particle-size fractionation on the original properties of sediment during transport present major challenges to tracing sediment provenance. In general, during transport the average size of particles decrease, while the degree of sorting and the average roundness increases with distance travelled. These changes result from a combination of selective transport and particle abrasion^49,58,59^ and often alter both the physical and chemical properties used to trace the source of the material. To assess whether our EoR and plume sediment samples have been affected by particle sorting we compared the major element concentrations in these samples with those from each of the major sub-catchments. Any changes in major element concentrations will also affect the concentrations of the trace elements.

For the Kruskal–Wallis H-test, conducted to test the ability of each element to distinguish between each of the source groups, a test statistic of p > 0.05 was used to exclude elements from further consideration following Collins et al.^35,60^ (Supp. Table 1). Elements which passed this test were then used in linear discriminant analysis to identify the best combination of elements which could classify each sample back to its original source (Supp. Table 2). The elements that could correctly classify the sources were then used in agglomerative-hierarchical-clustering to identify the closest geochemical associations of the EoR samples.

Agglomerative hierarchical clustering was implemented in the software package StatistiXL (version 2 2024). Ward’s clustering criteria was used to hierarchically consolidate normalized concentration values from samples into successively larger groups^61^ (Supp. Figure 1). This was done using both the individual sample concentrations for each element which passed both the Kruskal–Wallis H-test and was used in the linear discriminant analysis (Supp. Table 2). Variable concentrations were normalized to values between 0 and 1, using the maximum and minimum values, prior to clustering.

#### Clay minerals

Similarly, quantification of the clay mineral composition of sediments can provide an additional line of evidence to assign sediment contributions to different rock types and catchment source areas^15^. It can also be used to identify particle-size fractionation processes and the preferential transport of certain clay minerals across both river catchments and the adjacent marine environment^44,56^.

### Surface or sub-surface erosion

In the Australian environment, ^137^Cs is a product of atmospheric nuclear weapons testing that occurred during the 1950–70 s. Initially the distribution of this nuclide in the soil decreased exponentially with depth, with the maximum concentration at the surface. However, due to processes of diffusion, the maximum concentration in undisturbed soils is now generally found just below the surface. The bulk of the activity of this nuclide is, however, still retained within the top 100 mm of the soil profile. As the fallout radionuclide is concentrated in the surface soil, sediments derived from sheet and rill erosion will have high concentrations of the nuclide, while those eroded from gullies or channels have little or no fallout nuclide present^62,63^. To determine the dominant erosion process generating the SPM, we compared ^137^Cs activity concentrations in samples collected from surface soils and sub-surface erosion sources with the concentrations in the SPM samples collected from the EoR and plumes. To characterise the ^137^Cs activity concentration in both the surface-soil and channel end members we have used data from Wilkinson et al.^26^, corrected for decay.

### Supplementary Information

Supplementary Information.

## Data Availability

All data generated and analysed during this study are available from the corresponding author on reasonable request.
