# Supplementary material for: Tracing sources of inorganic suspended particulate matter in the Great Barrier Reef lagoon, Australia
Source: Sci Rep. 2024 Jul 8;14:15651. doi: 10.1038/s41598-024-66561-5 (PMC11231178; doi:10.1038/s41598-024-66561-5)
Supplement: Supplementary file 1 — Supplementary Information. [file 41598_2024_66561_MOESM1_ESM.docx]

Tracing sources of inorganic suspended particulate matter in the Great Barrier Reef lagoon, Australia. Zoe T. Bainbridge et al., Supplementary material.

**Supplementary Material: Determining the provenance of sediments**

**Kruskal–Wallis H-test:** To identify the sediment source of the Burdekin EoR samples we compared their geochemistry to a suite of samples collected from the six major Burdekin sub-catchments (Upper Burdekin n=22, Belyando n=10, Cape n=8, Suttor n=13, Bowen n=11, Bogie n=7). The nonparametric Kruskal–Wallis H-test was first used to test the ability of each element to distinguish between each of the sediment source groups, a test statistic of p > 0.05 was used to exclude elements from further consideration following Collins et al. (1998, 2010). The results are reported in Supplementary Table 1.

**Supplementary Table 1:** Individual element Kruskal–Wallis H-test results for sediment source of samples collected from the six major Burdekin sub-catchments.

| Element | adjusted  H: | P value: |
| --- | --- | --- |
| MgO | 15.5 | 0.01 |
| Na_2_O | 19.3 | 0.00 |
| Eu | 23.0 | 0.00 |
| Al_2_O_3_ | 23.0 | 0.00 |
| Sc | 23.8 | 0.00 |
| Fe_2_O_3_ | 27.5 | 0.00 |
| TiO_2_ | 33.3 | 0.00 |
| Sr | 33.8 | 0.00 |
| Er | 36.0 | 0.00 |
| Ho | 36.2 | 0.00 |
| Gd | 36.5 | 0.00 |
| Sm | 36.8 | 0.00 |
| Nd | 37.2 | 0.00 |
| Lu | 37.3 | 0.00 |
| Yb | 37.4 | 0.00 |
| K_2_O | 37.9 | 0.00 |
| Dy | 38.8 | 0.00 |
| Tb | 39.0 | 0.00 |
| Ba | 39.1 | 0.00 |
| Pr | 40.0 | 0.00 |
| Ni | 42.1 | 0.00 |
| Th | 43.7 | 0.00 |
| Ce | 44.0 | 0.00 |
| La | 44.5 | 0.00 |
| Cr | 46.1 | 0.00 |
| Co | 46.7 | 0.00 |
| U | 47.4 | 0.00 |
| Y | 49.1 | 0.00 |
| Rb | 50.9 | 0.00 |
|  |  |  |
| CaO | 9.6 | 0.09 |
| SiO_2_ | 10.4 | 0.07 |
| Tm | 10.9 | 0.05 |

**Linear discriminant analysis:** Elements which passed the nonparametric Kruskal–Wallis H-test were then used in linear discriminant analysis to identify the best combination of elements which could classify each sample back to its original source. The combination of K_2_O, TiO_2_, Ce, Co, Cr, La, Th, Y, Ni, Rb, Ba, Dy, Pr, and Er were able to classify 100% of the source samples back to the six source sub-catchments (Supplementary Table 2).

**Supplementary Table 2:** Classification results for samples collected to characterise the major sediment source areas in the Burdekin catchment. The software package StatistiXL (version 2 2024) was used for this analysis.

| Actual Group | Predicted Group | | | | | | Correctly  Classified |
| --- | --- | --- | --- | --- | --- | --- | --- |
|  | Upper Burdekin | Cape | Belyando | Suttor | Bowen | Bogie |  |
| Upper Burdekin | 22 | 0 | 0 | 0 | 0 | 0 | 100% |
| Cape | 0 | 8 | 0 | 0 | 0 | 0 | 100% |
| Belyando | 0 | 0 | 10 | 0 | 0 | 0 | 100% |
| Suttor | 0 | 0 | 0 | 13 | 0 | 0 | 100% |
| Bowen | 0 | 0 | 0 | 0 | 11 | 0 | 100% |
| Bogie | 0 | 0 | 0 | 0 | 0 | 7 | 100% |
| Overall Correct Classification Rate | | |  |  |  |  | 100% |

**Agglomerative-hierarchical-clustering:** The combination of K2O, TiO2, Ce, Co, Cr, La, Th, Y, Ni, Rb, Ba, Dy, Pr, and Er were then used (after normalisation of the concentrations) in agglomerative-hierarchical-clustering to identify the closest geochemical associations of the EoR samples. Variable concentrations were normalized to values between 0 and 1, using the maximum and minimum values, prior to clustering. The 2017 EoR sample most closely matched samples from the Bowen River (Supplementary Figure 1). All the 2018 EoR samples most closely matched samples collected from the Upper Burdekin sub-catchment. Agglomerative hierarchical clustering was implemented in the software package StatistiXL (version 2 2024). Ward’s clustering criteria was used to hierarchically consolidate normalized concentration values from samples into successively larger groups (Ward, 1963).


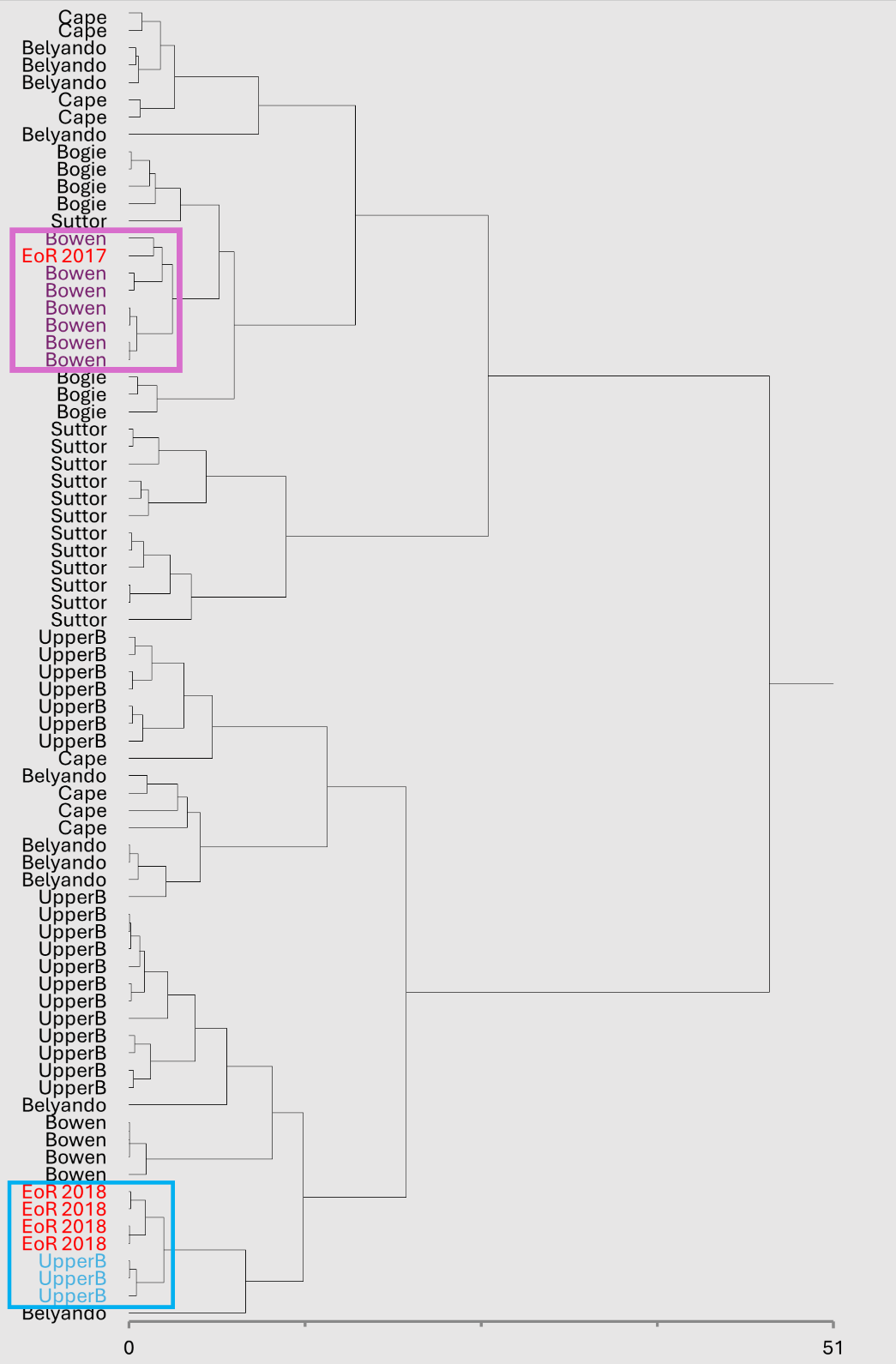


**Supplementary Figure 1:** Agglomerative hierarchical clustering results for the 2017 and 2018 EoR and source area samples from the Burdekin catchments. The bottom scale indicates the degree of similarity. The lower the axis of the dendrogram sample branches join the more similar they are.
